# Study on the effect of negative pressure occlusion drainage combined with silver ion dressing on inflammatory factors (IL-6, TNF-α) and healing effect of diabetic foot ulcer

**DOI:** 10.3389/fendo.2025.1689232

**Published:** 2025-11-28

**Authors:** Lu Wang, Sheng-Feng Gao, Ya-Su Jiang, Peng Chen, Zhen-Hua Gong

**Affiliations:** Department of Burn and Plastic Surgery and Wound Repair, Nantong First People’s Hospital, Nantong, Jiangsu, China

**Keywords:** diabetic foot, negative pressure closed drainage, silver ion dressings, inflammatory factors, ulcers

## Abstract

**Background:**

Diabetic foot ulcers impair quality of life and prognosis in diabetes. IL-6 and TNF-α regulate wound healing through inflammation. Although negative pressure wound therapy and silver dressings aid chronic wound repair, their combined efficacy in DFUs remains understudied.

**Objective:**

This study aimed to assess how negative pressure occlusion drainage combined with silver ion dressing affects inflammatory cytokine levels (IL-6, TNF-α) and wound healing in patients with diabetic foot ulcer.

**Methods:**

This study included 78 DFU patients treated with NPWT plus silver dressings (February 2023-April 2025). Serum IL-6 and TNF-α levels were measured at baseline, day 14, and day 30 post-treatment.

**Results:**

The findings indicated that IL-6 and TNF-α levels were significantly reduced at 14- and 30-days post-treatment compared to pre-treatment levels (P < 0.001), and the findings indicated a notable reduction in the wound area, with a healing duration of 19.49 ± 4.18 days and granulation tissue appearing in 6.83 ± 1.85 days. After 30 days of treatment, the healing rate reached 93.59%. The incidence of adverse reactions was low, 6.41%, and most of them were mild skin itching, nausea and vomiting, and a small amount of diarrhea.

**Conclusion:**

Negative pressure occlusion drainage combined with silver ion dressing has potential advantages in reducing inflammatory response and promoting the healing of diabetic foot ulcers, which is worthy of clinical application. In the future, multicenter randomized controlled trials are needed to further verify its efficacy and safety.

## 1 Introduction

Type 2 diabetes mellitus is a common endocrine and metabolic disease in clinical practice, which causes vascular and nerve damage during the course of the disease, causing a variety of complications. Diabetic foot ulcer is one of the common and serious complications of diabetes mellitus, and its incidence is increasing worldwide, which has a profound impact on the quality of life and survival prognosis of patients (1, 2). The pathophysiology of diabetic foot ulcers is complex and involves neuropathy, trauma, and, in many patients, peripheral arterial disease (3, 4). Diabetic foot ulcers are not only an important cause of hospitalization and amputation, but also a high mortality rate (5–7).

Currently, the traditional treatments for diabetic foot in clinical practice include glycemic control, local debridement, infection control, and dressing change, for ulcers that are difficult to heal or slow to heal, healing of ulcer wounds is not satisfactory (8, 9).In recent years, negative pressure closed drainage has been widely used in complex wound management, which significantly improves the wound healing environment by promoting blood circulation, reducing edema, removing metabolites and reducing infection through continuous or intermittent negative pressure (10, 11).It has been reported that the use of closed negative pressure drainage can promote wound healing in fractures and soft tissue injuries (12). Silver ion dressings play an important role in wound management due to their excellent antimicrobial properties, especially in the treatment of diabetic foot ulcers. Studies have shown that silver ion dressings can effectively reduce the bacterial burden on wounds and promote wound healing (13). In the treatment of diabetic foot ulcers, silver ion dressings significantly accelerate the wound healing process by reducing bacterial growth and preventing the invasion of external bacteria (14). The study found that silver ions effectively inhibit bacterial biofilm formation, crucial for healing chronic wounds resistant to conventional treatments. Silver dressings enhance wound healing without toxicity and promote healing in diabetic foot ulcers by regulating inflammatory factors like IL-6 and TNF-α (15).

However, research on combining negative pressure drainage with silver ion dressings for diabetic foot ulcers is limited, particularly regarding its impact on inflammation and healing time. Therefore, the purpose of this study was to observe the potential benefits of negative pressure occlusion drainage combined with silver ion dressing in improving inflammatory status and promoting diabetic foot ulcer healing through retrospective analysis, so as to provide a basis for further optimization of this treatment strategy.

## 2 Materials and methods

### 2.1 General information

From February 2023 to April 2025, 78 patients with diabetic foot ulcers were treated with negative pressure occlusion drainage and silver ion dressings at our hospital. The diagnosis of diabetic foot infection was based on the standardized treatment protocol (16), and ulcers were classified according to the Wagner grading system (17).

### 2.2 Inclusion and exclusion criteria

Inclusion criteria: (1) Diagnosis of diabetic foot infection according to established guidelines (16). (2) Ulcer grade 1–4 on the Wagner scale (18). (3) Treatment with negative pressure occlusion drainage combined with silver ion dressing; (4) Complete clinical data available.

Exclusion Criteria: (1) Type 1 diabetes; (2) Severe systemic diseases, malignancies, or hematologic disorders; (3) Malignant transformation of the ulcer; (4) Active infection at other sites; (5) Incomplete clinical data.

### 2.3 Ethics approved

This study was reviewed and approved by the Ethics Committee of Nantong First People’s Hospital (Approval No.: 2025KT111). The requirement for informed consent was waived due to the retrospective nature of the study.

### 2.4 Treatment protocol

Negative pressure occlusion drainage was performed using a vacuum-assisted closure (VAC) system (KCI, USA) with a continuous negative pressure of –125 mmHg. The dressing was changed every 3–5 days. Silver ion dressing (Acticoat™, Smith & Nephew) with sustained release of silver ions at a concentration of 70–100 μg/cm² was applied directly to the wound bed after each debridement.

### 2.5 Wound assessment and follow-up

Wound area was measured using a transparent film tracing method and calculated as length × width. Assessments were performed at baseline, day 14, and day 30. Granulation tissue appearance and healing time were recorded. Serum levels of IL-6 and TNF-α were measured using enzyme-linked immunosorbent assay (ELISA) kits (R&D Systems, USA) according to the manufacturer’s instructions. All assays were performed in duplicate according to the manufacturer’s instructions, and the mean values were used for analysis. The intra- and inter-assay coefficients of variation were <10%.

### 2.6 Data collection

Baseline demographic and clinical data were collected from electronic health records, including age, sex, body mass index (BMI), ulcer location, area, duration, Wagner grade, history of diabetes, glycated hemoglobin (HbA1c), fasting plasma glucose (FPG), blood pressure, and comorbidities such as hypertension, retinopathy, peripheral neuropathy, and renal disease. BMI was derived from the formula: weight (kg)/height (m²).

### 2.7 Outcome measures

Ulcer-related indicators: Ulcer area before and after treatment.

Ulcer healing status: The time of ulcer healing after treatment and the time of appearance of granulation tissue on the ulcer surface.

Micro-inflammation indicators in ulcer area: IL-6 and TNF-α levels before and after treatment.

Criteria for judging clinical efficacy: According to the clinical symptoms and Wagner grading indexes, it is divided into cured, effective, effective and ineffective. Total effective rate = (cure + effective + effective)/total number of cases× 100%.

Adverse reactions: Mainly itchy skin, nausea, vomiting and diarrhea.

### 2.8 Statistical analysis

Statistical analysis was performed using SPSS 26.0 (IBM, USA). Continuous variables are expressed as mean ± standard deviation, and categorical variables as percentages. Paired t-tests were used for within-group comparisons. A P-value < 0.05 was considered statistically significant. Graphs were generated using GraphPad Prism 7.0.

## 3 Results

### 3.1 Baseline information

Among the 78 patients with diabetic foot ulcers included in this study, 65.38% (51) were males and 34.62% (27) were females. The average age of the patients was 50.73 ± 8.39 years, and the average duration of diabetes was 10.62 ± 6.48 years. The average BMI was 24.73 ± 3.26 kg/m². In terms of glycemic control, the average HbA1c and FPG were 8.23 ± 0.87%, and the FPG was 10.27 ± 1.81 mmol/L. The average ulcer duration was 1.96 ± 0.95 months, and the average ulcer area was 508.36 ± 10.08 mm².

**Figure 1:**
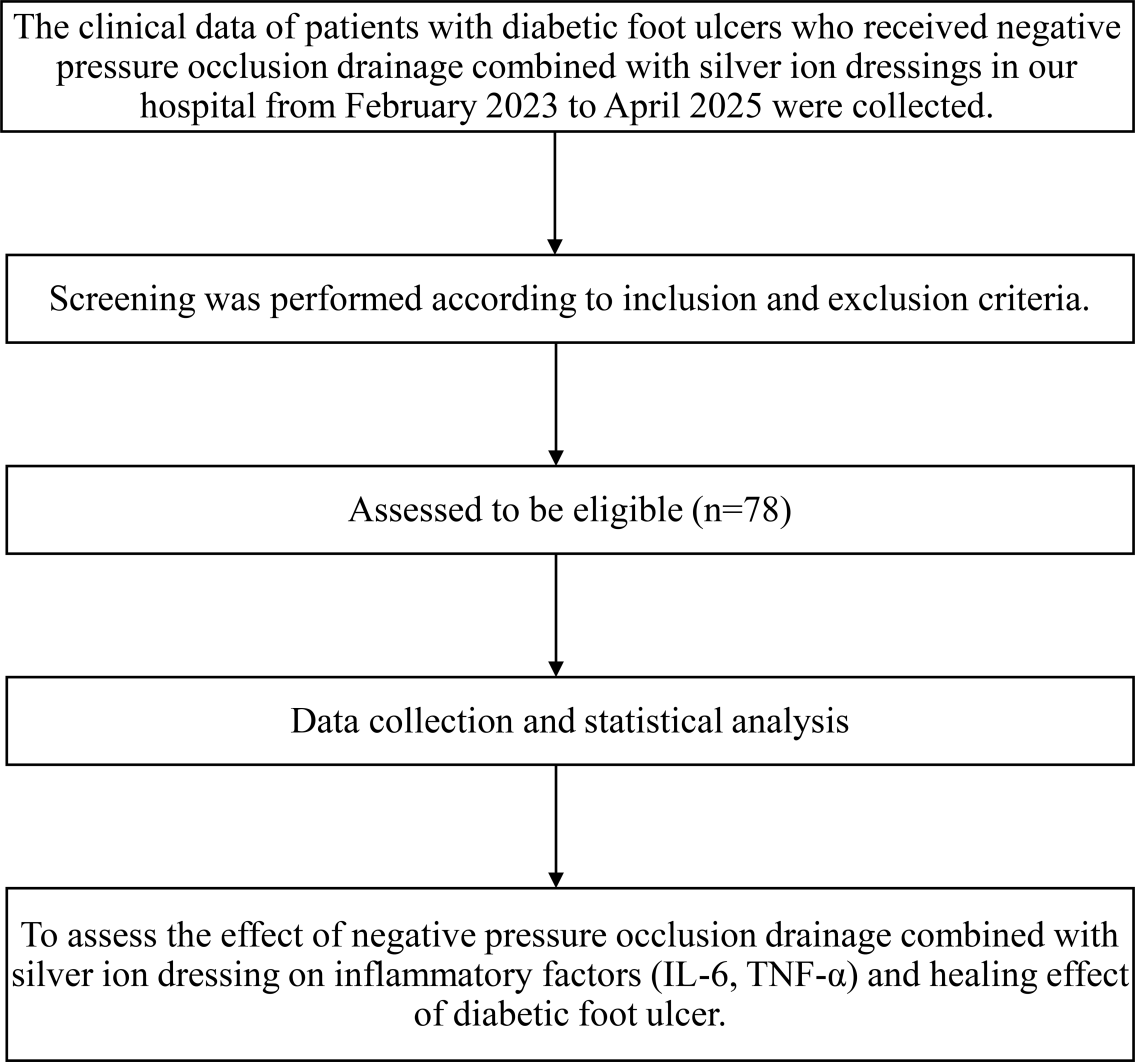
Patient Inclusion flow chart.

Regarding Wagner grade distribution, there were 3 patients (3.85%) with grade I, 40 patients (51.28%) with grade II, 14 patients (17.95%) with grade III, and 21 patients (26.92%) with grade IV. Common comorbidities included hypertension (25 cases, 32.05%), retinopathy (31 cases, 39.74%), peripheral neuropathy (23 cases, 29.49%), and kidney disease (25 cases, 32.05%). In addition, 39 patients (50.00%) had used anti-infective drugs in the past 3 months. The specific information is shown in **Table 1**, and the inclusion flow chart is shown in **Figure 1**.

**Table 1:** Clinical indexes of diabetic foot ulcer patients.

Index	Number of patients (n = 78)	Percentage (%)
Sex		
Male	51	65.38
Female	27	34.62
Age (years)	50.73 ± 8.39	
Diabetes duration (years)	10.62 ± 6.48	
BMI (kg/m^2^)	24.73 ± 3.26	
HbA1c (%)	8.23 ± 0.87	
FPG (mmol/L)	10.27 ± 1.81	
Ulcer course (months)	1.96 ± 0.95	
Ulcer size(mm^2^)	508.36 ± 10.08	
Wagner Grade		
I	3	3.85
II	40	51.28
II	14	17.95
IV	21	26.92
Comorbidities		
Hypertension	25	32.05
Retinopathy	31	39.74
Peripheral neuropathy	23	29.49
Kidney disease	25	32.05
Use of antimicrobial drugs within the last 3 months	39	50.00

BMI, Body mass index; HbA1c, Hemoglobin A1c; FPG, Fasting plasma glucose.

Patients may have more than one comorbidity. Percentages for comorbidities are based on the total number of patients (n=78).

### 3.2 Comparison of ulcer area before and after treatment

**Figure 2:**
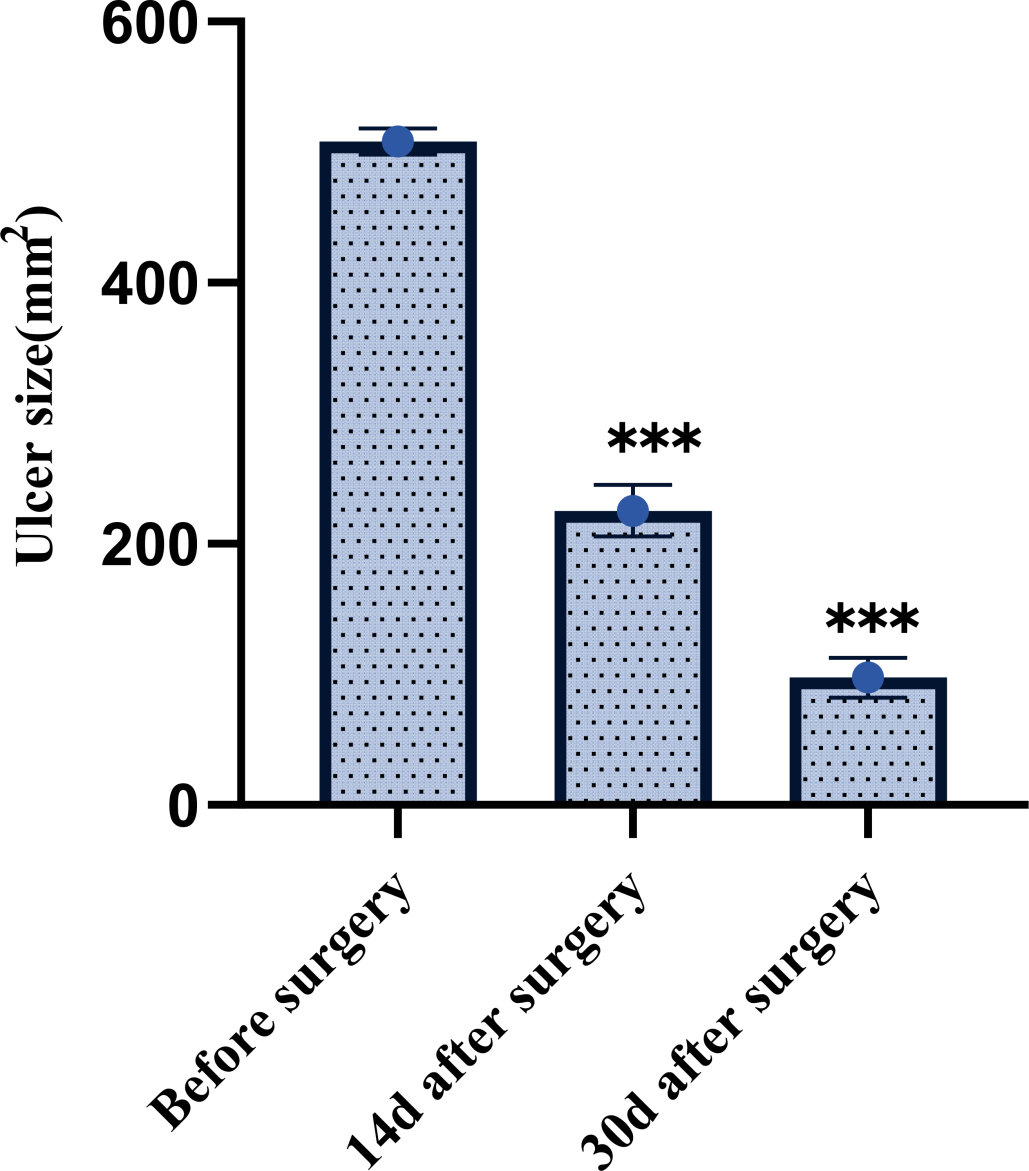
Comparison of ulcer area before and after treatment in patients with diabetic foot ulcers. ***P < 0.001.

The mean ulcer area was 508.36 ± 10.08 mm² before treatment. Following 14 days of treatment, the area decreased to 225.32 ± 19.75 mm², and further reduced to 97.64 ± 15.29 mm² after 30 days. The reduction in ulcer area was statistically significant at both post-treatment time points compared to baseline (P < 0.001). This is shown in **Figure 2**.

### 3.3 Time to ulcer healing and time to appearance of granulation tissue

**Figure 3:**
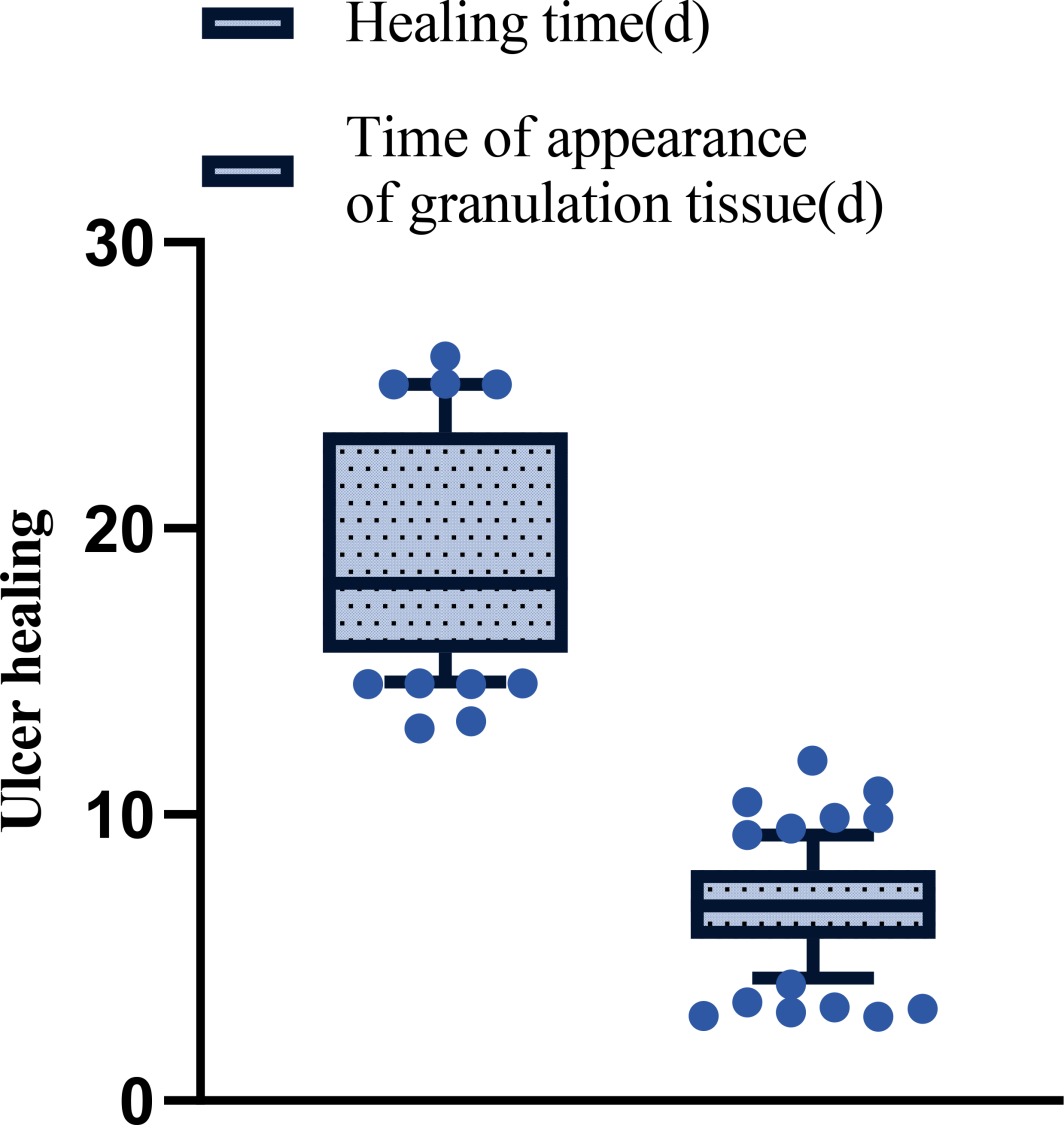
The patient’s ulcer healing time and time of appearance of granulation tissue after treatment.

The ulcer healing time and the time to appearance of granulation tissue were evaluated by the same blinded clinician who performed the wound assessments. After treatment, the ulcer healing time across all 78 patients was 19.49 ± 4.18 days, and the appearance time of granulation tissue on the ulcer surface was 6.83 ± 1.85 days. No patients were excluded from this analysis, and these values represent the cohort’s average healing trajectory. See **Figure 3**.

### 3.4 IL-6 and TNF-α levels before and after treatment

Before treatment, the average serum levels of IL-6 and TNF-α were 16.92 ± 1.43 ng/L, and those of TNF- were 18.91 ± 2.64 ng/L. On the 14th day of treatment, the levels of IL-6 and TNF-α decreased significantly to 9.57 ± 1.12 ng/L, and the TNF- decreased to 11.35 ± 1.83 ng/L, which was statistically significant compared with that before surgery (P < 0.001). By the 30th day of treatment, the IL-6 level was further reduced to 4.16 ± 0.68 ng/L, and the TNF-α was reduced to 7.13 ± 0.86 ng/L, which also showed a significant difference compared with the pre-treatment (P < 0.001). This is shown in **Table 2**. These results showed that the level of inflammatory cytokines decreased significantly during treatment.

**Table 2:** Comparison of inflammatory factors before and after treatment.

Index	IL-6 (ng/L)	TNF-α (ng/L)
Preoperative	16.92 ± 1.43	18.91 ± 2.64
Postoperative 14 d	9.57 ± 1.12	11.35 ± 1.83
Postoperative 30 d	4.16 ± 0.68	7.13 ± 0.86
P	<0.001	<0.001

IL-6: interleukin 6; TNF- α: tumor necrosis factor-α.

### 3.5 Effectiveness of ulcer healing

After 30 days of treatment, 21 of the 78 patients were healed (cured), accounting for 26.92% of the total. There were 43 cases with obvious effects, accounting for 55.13%; There were 9 cases with average effect, accounting for 11.54%; There were 5 cases with no obvious effect, accounting for 6.41%. The overall effective rate of treatment (including those with healing, obvious effect and efficacy) reached 73 cases, accounting for 93.59% of the total cases, indicating that the combination treatment had high clinical effectiveness. This is shown in **Table 3**.

**Table 3:** Patients have an effective rate of ulcer healing after treatment, n (%).

Index	Number of patients(n=78)	Percentage (%)
Cure	21	26.92
Obvious effect	43	55.13
Effective	9	11.54
Ineffective	5	6.41
Total Effect Rate	73	93.59

### 3.6 Adverse effects

Fewer adverse reactions were observed during the treatment period and the incidence was lower. Specifically, there were 2 cases (2.56%) of skin itching, 2 cases of nausea and vomiting (2.56%), and 1 case of diarrhea (1.28%). All adverse reactions were mild and did not affect the patient’s continued treatment or prognosis after symptomatic management. The total number of adverse reactions was 5 cases, accounting for 6.41% of the total cases, indicating that the overall safety of this treatment regimen was high, as shown in **Table 4**.

**Table 4:** Incidence of adverse reactions.

Index	Number of patients(n=78)	Percentage (%)
Itchy skin	2	2.56
nausea, vomiting	2	2.56
diarrhoea	1	1.28
Total number of cases	5	6.41

## 4 Discussion

In this study, we reported the effect of negative pressure occlusion drainage combined with silver ion dressing in patients with diabetic foot ulcers, and confirmed that it has significant advantages in reducing inflammatory response, shortening healing time, and improving healing rate. The study data showed that the levels of inflammatory cytokines IL-6 and TNF-α decreased significantly during the treatment process, accompanied by the continuous reduction of wound area and the obvious acceleration of healing speed, which further verified the potential mechanism of this combined treatment regimen in promoting wound repair.

Regarding the regulation of inflammatory response, it has been pointed out that a controlled inflammatory response is necessary for wound healing, but excessive inflammatory response can delay healing and even lead to worsening ulcers (19). The inflammatory response is crucial for wound healing, as moderate inflammation clears pathogens and dead tissue, setting the stage for recovery. However, excessive or prolonged inflammation can cause tissue damage, delay healing, and lead to chronic ulcers (20). This study found that inflammatory factors significantly decreased 30 days post-surgery, indicating effective inflammation suppression and reduced tissue damage, aligning with previous findings on closed drainage (21). Modulating inflammation is vital for healing, with microRNAs like miR-19a/b and miR-20a aiding keratinocyte response, and the CD44 pathway enhancing recovery post-myocardial infarction by regulating inflammation and fibrosis (22). The CD44 signaling pathway is crucial for post-myocardial infarction healing by modulating inflammation and fibrosis (23). Effective chronic wound treatment involves reducing inflammation and promoting angiogenesis through chemokine modulation (24). Shifting macrophages from a pro-inflammatory (M1) to a healing (M2) state enhances tissue repair (25).

Ulcer areas decreased from 508.36 mm² to 97.64 mm² after 30 days, with an average healing time of 19.49 days, demonstrating that combining negative pressure drainage and silver ion dressing significantly improves blood circulation and wound healing (26). The analysis is probably due to the fact that silver ions have wide-ranging antimicrobial properties, allowing them to effectively curb bacterial growth and thus reduce infection risk. When these two techniques are combined, a synergistic effect can be created, further reducing the healing time and granulation tissue growth time in patients with diabetic foot ulcers, which is consistent with previous studies (27).Notably, the overall healing rate in this study was 93.59%, showing the clinical potential of the combination therapy. Another study explored the effects of autologous platelet-rich plasma (PRP) in combination with negative pressure occlusion drainage in pressure ulcer repair, and the results showed that this combination method can significantly improve clinical outcomes, reduce inflammation, reduce pain, accelerate wound healing, and reduce complication rates (28). Although there was no control group in this study, combined with the results of previous multicenter randomized controlled studies, the application effect of this regimen in refractory diabetic foot ulcer is worthy of clinical promotion.

In addition, fewer adverse reactions were observed during the treatment and the incidence was low, indicating that the regimen was safe. Mild skin itching, nausea, vomiting, and diarrhea were common side effects, and there were no significant disabling or treatment-affecting adverse events, which supported its safety.

However, there are limitations to this study. First, as a retrospective single-center study, the sample size has reached 78 cases, but a larger-scale, multicenter prospective randomized controlled trial is still needed to verify the generalizability of its efficacy. Second, the lack of a control design of the control group limits the clear inference of causality, and future studies should add a control group to validate the advantages of the combination regimen. In addition, there are insufficient data on long-term follow-up to judge the effect of this regimen on recurrence rates and long-term quality of healing.

## 5 Conclusion

In summary, negative pressure occlusion drainage combined with silver ion dressing has shown good clinical effects in reducing inflammatory response, shortening healing time and improving healing rate, and has the potential to be popularized. In the future, multicenter, randomized controlled clinical trials should be carried out to further optimize the treatment regimen and confirm its efficacy and safety in different patient populations, so as to provide stronger evidence for the clinical management of diabetic foot ulcers.

## Data Availability

The original contributions presented in the study are included in the article/supplementary material. Further inquiries can be directed to the corresponding author.
